# Prevalence of sedentary behavior and its correlates among primary and secondary school students

**DOI:** 10.1016/j.rppede.2015.09.002

**Published:** 2016

**Authors:** Rodrigo Wiltgen Ferreira, Airton José Rombaldi, Luiza Isnardi Cardoso Ricardo, Pedro Curi Hallal, Mario Renato Azevedo

**Affiliations:** a Physical Education Post Graduation Program, Universidade Federal de Pelotas (Ufpel), Pelotas, RS, Brazil; b Epidemiology Post Graduation Program, Universidade Federal de Pelotas (Ufpel), Pelotas, RS, Brazil

**Keywords:** Sedentary lifestyle, Adolescent behavior, Adolescents, Television, Internet

## Abstract

**Objective:**

To determine the students’ exposure to four different sedentary behavior (SB) indicators and their associations with gender, grade, age, economic status and physical activity level.

**Methods:**

A cross-sectional study was conducted in 2013. The SB was collected using the HELENA instrument, composed by screen time questions (TV, video games and internet) and sitting activities on school opposite shift. The cut point of ≥2h/day was used to categorize the outcome. The Poisson regression was used for associations between the outcome and the independent variables (95% significance level), controlling for confounding variables and the possible design effect.

**Results:**

The sample was composed by 8661 students. The overall prevalence of SB was 69.2% (CI95% 68.1–70.2) on weekdays, and 79.6% (CI95% 78.7–80.5) on weekends. Females were more associated with the outcome, except to electronic games. Advanced grades students were more involved in sitting tasks when compared to the early grades. Older students were more likely to surf on net for ≥2h/day. Higher economic level students were more likely to engage in video games and internet. Active individuals were less likely to engage in SB on weekdays.

**Conclusions:**

The prevalence of SB was high, mainly on weekends. The associations with sex, age, grade and physical activity level should be considered into elaboration of more efficient interventions on SB control.

## Introduction

Since the end of World War II, there was an intensification in the communication process, particularly stimulated by television watching. There are several benefits of intensifying the communication process, but in recent decades studies have shown that excessive sedentary time can lead to poor health, particularly among the new generations that grow in an era of massive technology use.^1^ Sedentary behavior (SB) is being conceptualized in the literature as any activity with an energy cost equal to or less than 1.5 METs,^1^ ^1^ held in reclining or sitting posture.^2^

Childhood and adolescence are particularly relevant for the study of SB because the period is characterized by marked physical and mental changes.^3^ In this sense, there is evidence that SB plays directly impacts on many health outcomes, such as obesity, metabolic syndrome and cardiovascular diseases,^4^–^6^ also been described as related to reductions in life expectancy.^7^ Due to its effects on health, recommendations on SB were released in 2001, with an update on 2011.^8^

A recent review study identified 24 Brazilian studies about SB, most of which focusing on digital media or screen time (television, games and computer).^9^ However, differences in measurement tools (questionnaire structure), as well as analytical approaches (SB thresholds, regression types, and possible confounder control) make it difficult to compare data from different studies. In addition, it is necessary to analyze the possible associations with social, demographic and behavioral variables in order to conduct effective interventions on controlling SB.

The aim of the present study was to evaluate exposure to four different indicators of SB among adolescents of Pelotas, Brazil, and its associations with gender, grade, age, economic level and physical activity.

## Method

This cross sectional study was part of the third follow-up data collection of an intervention called “*Physical Education* +: *Practicing health at school*”. This study was conducted in 56 public schools of the city of Pelotas, Brazil in 2012 and 2013. The main objective of the intervention was to disseminate information related to physical activity and general health through physical education classes. Data presented in this article are a snapshot of exposure to SB.

A multistage sampling process was used, divided on two steps, referring to each intervention year. Each year it was conducted a raffle among all the city eligible schools to guarantee the representativeness of the sample. More information about the sampling process is available on the Spohr et al.^10^ paper. The first step was conducted on 2012. A list of the primary and secondary public schools of the city was obtained. We then stratified school according to type (state vs. city) and city area (urban vs rural). A random strategy was adopted to selected school in each strata, totaling 40 schools in the sample on 2012. On the second year of the study (2013) the same sampling strategy was adopted, but another 18 schools were included. Two schools were removed from the original sample (raffled on 2012). One school refused to participate on the study, and another was excluded because all the eligible students belong to the night shift. An important point is that after the data collection beginning on 2013 there was no other refuses. The final number of participating schools (n=56) represented 67% of all the eligible schools on the city.

An adapted version of the “HELENA” instrument, first proposed by Rey-López et al.^11^ (Kappa coefficient >0.7), was used to assess SB. The instrument was translated into Portuguese and then back to Spanish, in order to ensure information clarity and meaning. SB is evaluated by questions about the use of television, electronic games, internet and academic activities on inverse shift classes. Questions are done first about week days and then about weekend days. To quantify the duration of SB, there is a time scale which the interviewee must choose between seven time categories, ranging from “none” to “four hours or more” per day. SB was categorized according to the recommendations of the American Academy of Pediatrics.^8^

The data collection occurred from 2013 March to May. Students from grades 5 to 12 were invited to participate of the study. The questionnaire was self-administered in the class room under trained interviewer supervision. The administration of the questionnaire was collective. The students filled up the questions after the interviewer's explanation for every question. If a student had any doubt, the interviewer should solve it individually.

The independent variables used in this analysis were sex, age (categorized in five groups <12, 13, 14, 15, ≥16), grade (5th to 12th grade), socioeconomic status and physical activity. The socioeconomic status classification was based on an assets index later categorized into quintiles, following principal component analysis. The questionnaire by Farias et al.^12^ was used to assess physical activity levels (validity: *k*=0.59 and CCI=0.88/reproducibility: *k*=0.52 and Spearman=0.62). This instrument has a list of physical activities, in which the interviewee should answer about the frequency and duration of the activities performed on the previous week. A total physical activity score was calculated and later categorized as meeting current recommendations of 300min per week or not.^13^ As an operational decision, only the leisure time physical activity section was used. The original instrument was tested in public schools of two close cities.

Data were double entered in EpiData 3.1 program and the analyses were performed using Stata 12.0. Poisson regression was used in the adjusted analysis to verify the association between each type of sedentary behavior and the independent variables, adjusting for confounders and the possible design effect. Besides that, on the adjusted analysis, all independent variables with a *p*-value >0.20 were excluded from the model, and a 95% significance level was adopted for the associations between the outcome and the exposures.

The study was approved by the Ethics Committee in Research of the Physical Education School of the Federal University of Pelotas under the protocol 039/2011. Written consent was requested from parents of students under 18 years of age, and directly from students aged 18 or more years.

## Results

The sample comprised 8661 students, representing 57.7% of all eligible individuals. Response rates were 47.6% in secondary school and 59.7% in primary school. Most participants were females (53.1%), attended primary school (76.8%), were younger than 12 years (28.6%) and were active in leisure time (57.5%). The total sedentary behavior prevalence was 69.2% on weekdays and 79.6% on weekends.

**Figure 1:**
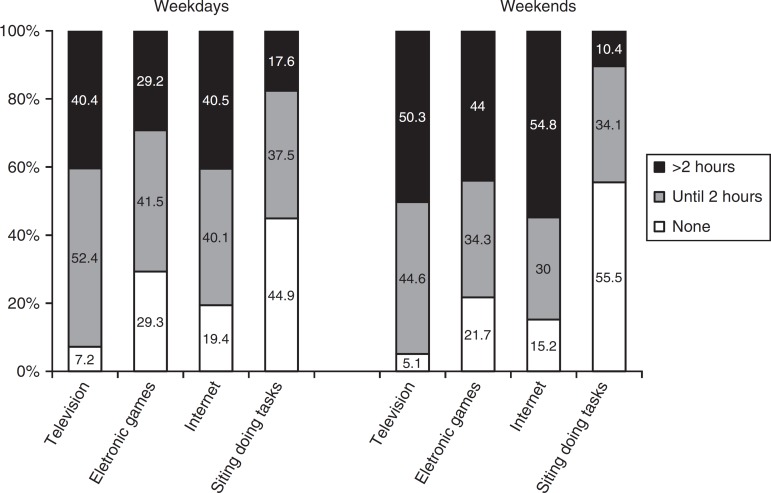
Students’ sedentary behavior on weekdays and weekends, Pelotas-RS Brazil, 2013, n=8661.

Fig. 1 describes the time used for each SB type on weekdays and weekend days. TV viewing for two or more hours per day was reported by 40% of the adolescents on weekdays and by 50% on weekend days. The proportion of students using electronic games for two or more hours per day was 29% on weekdays and 44% on weekend days. For internet use, these proportions were 41% and 55%, respectively. Spending two or more hours doing sitting-tasks at the inverse school shift was reported by 18% of the respondents on weekdays and by 10% on weekend days.

**Figure 2:**
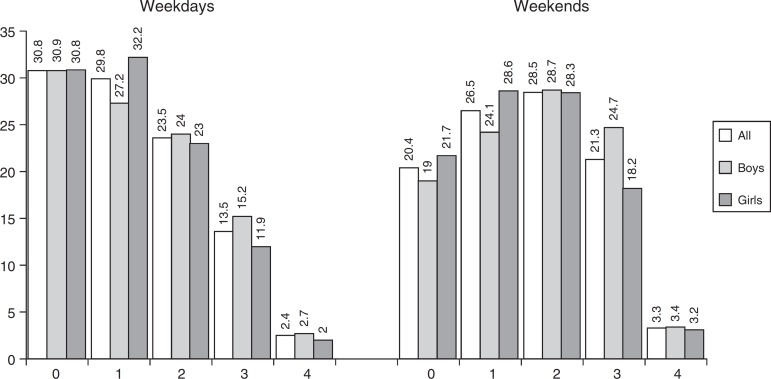
Accumulation of sedentary behavior on two or more hours per day in different indicators of sedentary behavior, Pelotas-RS Brazil, 2013, n=8661.

Fig. 2 illustrates the accumulation of SB for ≥2h/day on different types of behaviors measured. For the weekdays period, 31% of the sample accumulate ≤2h/day in any measured SB, 30% reported 2h or more per day in one behavior, 23% accumulated in two SB types, 14% in three types and 2% in the four types measured. On weekends the proportions were 20%, 27%, 29%, 21% and 3%, respectively.

Table 1 shows the adjusted analysis between excess of SB (≥2h/day) on weekdays according to the independent variables. Girls were more likely than boys to be watch TV for longer periods, as well as to perform sitting tasks at the inverse school shift. Boys, on the other hand, were more likely than girls to spend two or more hours per day playing electronic games. No sex differences were found for computer use. Performing sitting tasks on the inverse school shift increased according to grade. The use of electronic games and internet for two or more hours per day was higher among high socioeconomic level adolescents as compared to those from lower socioeconomic groups. Active adolescents were less likely to watch TV or use the internet for two or more hours per day as compared to their inactive peers.

**Table 1:** Adjusted analysis between excess of sedentary behavior (≥2h/day) and sociodemographic and behavioral variables on weekdays, Pelotas-RS Brazil, 2013, n=8661.

			Sedentary behavior ≥2h on weekdays														
			Television				Electronic games				Internet				Siting doing tasks		
			%	PR (CI95%)	*p* -value		%	PR (CI95%)	*p* -value		%	PR (CI95%)	*p* -value		%	PR (CI95%)	*p* -value
**Sex**					0.01				<0.001				0.36				0.005
	*Male*		38.3	1.0			37.9	1.0			39.3				16.2	1.0	
	*Female*		42.2	1.09 (1.01–1.18)			21.5	0.58 (0.54–0.63)			39.7				18.9	1.14 (1.03–1.26)	

**Grade**					0.09				<0.001				0.67				<0.001
	*Primary school*																
		5^a^	38.1	1.0			24.4	1.0			25.6				12.9	1.0	
		6^a^	39.5	0.99 (0.89–1.12)			28.1	1.11 (0.98–1.24)			32.2				13.3	0.98 (0.82–1.18)	
		7^a^	44.0	1.11 (0.99–1.24)			33.2	1.30 (1.12–1.52)			41.9				14.9	1.03 (0.84–1.27)	
		8^a^	41.9	0.99 (0.88–1.11)			34.3	1.31 (1.14–1.52)			48.4				20.6	1.38 (1.11–1.72)	
	*Secondary School*																
		1^a^	38.7	0.91 (0.80–1.03)			31.9	1.16 (0.96–1.40)			52.0				22.3	1.50 (1.18–1.92)	
		2^a^	39.3	0.93 (0.81–1.08)			26.7	1.02 (0.84–1.23)			50.7				28.4	1.84 (1.43–2.37)	
		3^a^	41.1	0.94 (0.77–1.13)			22.3	0.88 (0.70–1.11)			49.8				30.8	1.89 (1.36–2.64)	

**Age**					0.07				0.001				<0.001				<0.001
	≤ *12*		38.5	1.0			26.3	1.0			28.6	1.0			12.2	1.0	
	*13*		39.7	0.99 (0.90–1.11)			30.3	1.02 (0.92–1.14)			38.7	1.33 (1.19–1.49)			16.4	1.24 (1.06–1.44)	
	*14*		45.1	1.17 (1.07–1.27)			31.5	1.03 (0.92–1.16)			44.8	1.51 (1.36–1.67)			18.4	1.25 (1.02–1.53)	
	*15*		38.9	1.05 (0.93–1.18)			33.5	1.11 (0.98–1.27)			45.8	1.55 (1.39–1.74)			17.4	1.06 (0.88–1.29)	
	≥ *16*		40.3	1.10 (0.99–1.23)			26.9	0.97 (0.82–1.14)			47.0	1.54 (1.40–1.69)			26.4	1.32 (1.04–1.68)	

**Asset Index (Quintiles)**					0.36				<0.001				<0.001				0.53
	*1 (lower)*		41.6				14.9	1.0			16.1	1.0			16.5		
	*2*		41.4				26.0	1.75 (1.48–2.08)			34.6	2.21 (1.91–2.56)			16.1		
	*3*		38.4				30.7	2.04 (1.76–2.38)			41.9	2.63 (2.25–3.06)			18.7		
	*4*		41.1				34.5	2.31 (1.98–2.70)			47.5	2.96 (2.59–3.39)			17.8		
	*5 (higher)*		39.6				39.0	2.53 (2.19–2.94)			55.9	3.47 (3.00–4.01)			18.9		

**Physical activity**					0.02				0.35				0.003				0.39
	< *300min*		42.7	1.0			26.1				41.0	1.0			18.5		
	≥ *300min*		38.6	0.93 (0.87–0.99)			31.5				39.0	0.93 (0.89–0.98)			17.2		

Table 2 illustrates the adjusted analysis between excess of SB (≥2h/day) on weekend days according to the independent variables. Girls were more likely than boys to spend two or more hours in all SB, except playing electronic games. Those from higher grades were less likely to perform sitting tasks on weekends. The positive associations between socioeconomic status and (a) playing electronic games and (b) using the internet were confirmed for weekend days. Physical activity levels did not predict any type of SB on weekends.

**Table 2:** Adjusted analysis between excess of sedentary behavior (≥2h/day) and sociodemographic and behavioral variables on weekends. Pelotas-RS Brazil, 2013, n=8661.

			Sedentary behavior ≥2h on weekends														
			Television				Electronic games				Internet				Siting doing tasks		
			%	PR (CI95%)	*p* -value		%	PR (CI95%)	*p* -value		%	PR (CI95%)	*p* -value		%	PR (CI95%)	*p* -value
**Sex**					0.007				<0.001				0.04				0.03
	*Male*		48.7	1.0			55.4	1.0			54.3	1.0			10.0	1.0	
	*Female*		51.7	1.06 (1.02–1.11)			33.9	0.63 (0.59–0.66)			55.2	1.04 (1.00–1.09)			10.8	1.16 (1.02–1.32)	

**Grade**					0.68				<0.001				0.83				0.04
	*Primary school*																
		5^a^	49.2				42.4	1.0			43.4				10.9	1.0	
		6^a^	50.6				47.5	1.09 (1.02–1.18)			51.0				9.2	0.85 (0.65–1.11)	
		7^a^	51.5				46.8	1.09 (1.00–1.19)			55.3				9.3	0.76 (0.56–1.02)	
		8^a^	50.5				44.2	1.02 (1.93–1.11)			61.3				9.9	0.77 (0.56–1.06)	
	*Secondary School*																
		1^a^	50.1				44.4	0.97 (0.86–1.09)			65.0				9.6	0.64 (0.45–0.90)	
		2^a^	50.1				38.6	0.85 (0.76–0.96)			65.2				13.8	0.81 (0.56–1.16)	
		3^a^	59.4				33.3	0.74 (0.65–0.84)			62.1				16.2	0.90 (0.63–1.29)	

**Age**					0.30				0.94				<0.001				<0.001
	≤ *12*		49.6				43.0				44.5	1.0			8.9	1.0	
	*13*		52.8				47.5				55.7	1.23 (1.15–1.31)			8.9	1.11 (0.87–1.40)	
	*14*		51.5				44.5				61.0	1.32 (1.24–1.40)			9.9	1.29 (1.03–1.63)	
	*15*		48.1				45.9				58.0	1.27 (1.20–1.35)			10.3	1.40 (1.05–1.85)	
	≥ *16*		48.8				39.4				60.9	1.29 (1.23–1.37)			14.5	1.96 (1.52–2.54)	

**Asset Index (Quintiles)**					0.45				<0.001				<0.001				0.52
	*1 (lower)*		51.7				27.0	1.0			26.8	1.0			10.0		
	*2*		50.3				41.3	1.54 (1.39–1.71)			48.9	1.84 (1.69–2.01)			10.0		
	*3*		48.5				44.4	1.65 (1.50–1.82)			59.1	2.20 (1.98–2.46)			10.6		
	*4*		52.1				51.0	1.91 (1.68–2.17)			64.9	2.40 (2.18–2.65)			10.7		
	*5 (higher)*		49.0				55.7	2.04 (1.85–2.24)			62.1	2.70 (2.42–3.02)			10.8		

**Physical activity**					0.21				0.43				0.44				0.14
	< *300min*		51.7				38.5				55.1				10.1	1.0	
	≥ *300min*		49.3				47.7				55.0				10.3	1.07 (0.94–1.22)	

## Discussion

This study aimed to evaluate exposure to SB in a city in southern Brazil, as well as to study its association with sex, grade, age, economic status and physical activity level. The difficulties encountered by the international and national literature in establishing the real magnitude of the SB and its associated factors, stem from different cultures/customs employed in research and SB routine of each population. Thus contributing to SB understanding and its associated factors is important for interventions to focus on regular population interaction with digital media instead of banning the technology from people's lives.

There was a clear upward trend in SB in weekend days as compared to weekdays. Data from a multicenter study conducted in seven European countries using the same instrument to access SB found the same relationship.^14^ However, a review study on SB in children^15^ and a study using accelerometry^16^ found associations on the inverse direction, pointing to uncertainty on the relationship between SB and weekends. Cultural, environmental and social standards inherent of each region or country can influence SB and perhaps explain the differences across studies.

Specifically about the indicators of SB, there is solid evidence regarding screen time. The prevalence of excessive time watching television in this study corroborates with other published studies.^17^^,^ 18 For the remaining screen-related behaviors, the prevalence found here was also similar to previous studies.^19^^,^ 20 In a review study conducted by Barbosa Filho et al.,^9^ a large variation in the prevalence of SB was detected across studies. In studies using a cutoff of 2h daily, the prevalence ranged from 32% in a study conducted in the city of Foz do Iguaçu to 88% in a study in Ouro Preto, MG.^9^

It was possible to identify a large accumulation of different types of SB for ≥2h/day, especially on weekend days. The increasing use of digital medias promoted cultural changes in the society.^1^ The easy access to electronic devices, the changing family environments and the lack of neighborhood safety make even more children remain reclusive in their homes, fact that can foster greater exposure to SB.^21^ Furthermore, the use of more than one electronic device simultaneously is becoming common among young people, increasing SB in this population.^22^^,^ 23

Girls was more likely to exceed the daily recommended amount of SB on both weekdays and weekends in almost all types of SB, except “playing electronic games”. The association between SB and sex is not yet a consensus in the literature.^6^^,^ 24 The study by Atkin and colleagues,^25^ which used accelerometry to measure SB, demonstrated that the frequent outdoor activities restriction imposed by parents was associated with an increase in their daughters’ sedentary time after one year of follow-up.

Age was positively associated with using the internet in our sample. A study that evaluated the compulsive youth use of internet demonstrated a direct relationship with age as well.^26^ The different ways to use the internet and the interactivity with daily life make it a versatile and culturally accepted practice, with a strong tendency to intensify even more in the near future.^27^

Specifically regarding associations between grade and types of SB, there was an inverse relationship between spending time on electronic games and being involved in sitting tasks on inverse school shift on weekdays. The increased responsibilities over the years can be a major factor in this relationship. The search for good performances in selection processes and a higher search for improvement to conquer a place on the labor market are common among teenagers,^28^ which may explain the reduction in available time for SB when there are activities in counter-turn.

As for the relationship between economic level and SB, there was a direct relationship among them on weekday and weekends for all the screen related behaviors. Our associations between socioeconomic status and SB were consistent with previous studies. The increased family purchasing power is an important facilitator of children and adolescents SB.^5^^,^ 19 ^,^ 29

A recent meta-analysis conducted by Pearson and coworkers^30^ summarized the results of 163 studies published since August 2013 about the association between SB and physical activity. The review showed an inverse, but of weak magnitude, association. Here we found inverse associations for TV viewing and internet using, around 7%.

The present study has some limitations that should be noted. Firstly, exposures and outcomes were based on self-report. The cross-sectional nature of the data makes it impossible to study the temporality of the associations between SB and physical activity. The relatively low response rate, particularly for secondary school, may also affect our results.

In conclusion, the prevalence of SB in this sample was high, especially on weekends. SB seems more evident in those from higher socioeconomic status and girls, with the exception of electronic games. In addition, individuals belonging to higher grades and ages seem to be more likely to involve in sedentary tasks in the inverse school shift, and to use internet. Future SB studies should take these results into account due to the influence of associated factors as age, sex, grade and physical activity on interventions’ strategies. In addition, it is necessary that health and education professionals understand the importance of alerting young people and their caregivers about the risks of an overly sedentary routine.

## Footnotes

1 Unit of physical activity intensity referred to basal metabolism. One MET is equivalent to 3.5 ml/kg/min. or 1 kcal/kg/h. For example, vigorous walking requires four times more energy than basal metabolism, therefore, 4 METs. The fact of being in a standing position requires two times more energy than basal metabolism (2 METs), thus, the individual is not considered on SB.

## References

[1] Barr-Anderson D.J., Sisson S.B. (2012). Media use and sedentary behavior in adolescents: what do we know, what
has been done, and where do we go?. Adolesc Med State Art Rev.

[2] Sedentary Behaviour Research Network (2012). Letter to the editor: standardized use of the terms “sedentary” and
“sedentary behaviours”. Appl Physiol Nutr Metab.

[3] Alberga AS, Sigal RJ, Goldfield G, Prud’homme D, Kenny GP (2012). Overweight and obese teenagers: why is adolescence a critical
period?. Pediatr Obes.

[4] Tremblay MS, LeBlanc AG, Kho ME, Saunders TJ, Larouche R, Colley RC (2011). Systematic review of sedentary behaviour and health indicators in
school-aged children and youth. Int J Behav Nutr Phys Act.

[5] Rezende LF, Rodrigues Lopes M, Rey-López JP, Matsudo VK, Luiz OC (2014). Sedentary behavior and health outcomes: an overview of systematic
reviews. PLOS ONE.

[6] Pate RR, Mitchell JA, Byun W, Dowda M (2011). Sedentary behaviour in youth. Br J Sports Med.

[7] Katzmarzyk PT, Lee IM (2012). Sedentary behaviour and life expectancy in the USA: a cause-deleted
life table analysis. BMJ Open.

[8] American Academy of Pediatrics, Council on Communications and Media (2011). Children, adolescents, obesity and the media. Pedriatrics.

[9] Barbosa VC, Campos W, Lopes AS (2014). Epidemiology of physical inactivity, sedentary behaviors, and
unhealthy eating habits among Brazilian adolescents: a systematic
review. Cien Saude Colet.

[10] Spohr CF, Fortes MO, Rombaldi AJ, Hallal PC, Azevedo MR (2014). Atividade física e saúde na educação física escolar: efetividade de um
ano do projeto “Educação Física +”. Rev Bras Ativ Fis Saude.

[11] Rey-López JP, Ruiz JR, Ortega FB, Verloigne M, Vicente-Rodriguez G, Gracia-Marco L (2012). Reliability and validity of a screen time-based sedentary behaviour
questionnaire for adolescents: the HELENA study. Eur J Public Health.

[12] Farias JC, Lopes AS, Mota J, Santos MP, Ribeiro JC, Hallal PC (2012). Validade e reprodutibilidade de um questionário para medida de
atividade física em adolescentes. Rev Bras Epidemiol.

[13] World Health Organization (2010). Global recommendations on physical activity for health.

[14] Rey-López JP, Vicente-Rodríguez G, Ortega FB, Ruiz JR, Martinez-Gómez D, De Henauw S (2010). Sedentary patterns and media availability in European adolescents: the
HELENA study. Prev Med.

[15] De Craemer M, De Decker E, De Bourdeaudhuij I, Vereecken C, Deforche B, Manios Y (2012). Correlates of energy balance-related behaviours in preschool children:
a systematic review. Obes Rev.

[16] Ramirez-Rico E, HIlland TA, Foweather L, Férnandez-Garcia E, Fairclough SJ (2013). Weekday and weekend patterns of physical activity and sedentary time
among Liverpool and Madrid youth. Eur J Sport Sci.

[17] Camelo LV, Rodrigues JF, Giatti L, Barreto SM (2012). Lazer sedentário e consumo de alimentos entre adolescentes
brasileiros: Pesquisa Nacional de Saúde do Escolar (PeNSE), 2009. Cad Saude Publica.

[18] Wells JC, Hallal PC, Reichert FF, Menezes AM, Araújo CL, Victora CG (2008). Sleep patterns and television viewing in relation to obesity and blood
pressure: evidence from an adolescent Brazilian birth cohort. Int J Obes (Lond).

[19] Dumith SC, Hallal PC, Menezes AM, Araújo CL (2010). Sedentary behavior in adolescents: the 11-year follow-up of the 1993
Pelotas (Brazil) birth cohort study. Cad Saude Publica.

[20] Farias JC, Lopes AS, Mota J, Hallal PC (2012). Prática de atividade física e fatores associados em adolescentes no
Nordeste do Brasil. Rev Saude Publica.

[21] Cillero IH, Jago R (2010). Systematic review of correlates of screen-viewing among young
children. Prev Med.

[22] Jago R, Sebire SJ, Gorely T, Cillero IH, Biddle SJ (2011). “I’m on it 24/7 at the moment”: a qualitative examination of
multi-screen viewing behaviours among UK 10-11 year olds. Int J Behav Nutr Phys Act.

[23] Ferrar K, Chang C, Li M, Olds TS (2013). Adolescent time use clusters: a systematic review. J Adolesc Health.

[24] Uijtdewilligen L, Nauta J, Singh AS, Van Mechelen W, Twisk JW, van der Horst K (2011). Determinants of physical activity and sedentary behaviour in young
people: a review and quality synthesis of prospective studies. Br J Sports Med.

[25] Atkin AJ, Corder K, Ekelund U, Wijndaele K, Griffin SJ, van Sluijs EM (2013). Determinants of change in children's sedentary time. PLOS ONE.

[26] Van Roji AJ, Schoenmakers TM, van de Eijnden RJ, van de Mheen D (2010). Compulsive Internet use: the role of online gaming and other Internet
applications. J Adolesc Health.

[27] Jungblut AL (2004). A heterogenia do mundo on-line: algumas reflexões sobre virtualização,
comunicação mediada por computador e ciberespaço. Horizontes Antropológicos.

[28] Lemos AE, Pinto MC (2008). Empregabilidade dos administradores: quais os perfis profissionais
demandados pelas empresas?. Cadernos Ebape Br.

[29] Verloigne M, Van Lippelvelde W, Maes L, Brug J, De Bourdeaudhuij I (2012). Family- and school-based correlates of energy balance-related
behaviours in 10–12-year-old children: a systematic review within the ENERGY
(EuropeaN Energy balance Research to prevent excessive weight Gain among Youth)
project. Public Health Nutr.

[30] Pearson N, Braithwaite RE, Biddle SJ, van Sluijs EM, Atkin AJ (2014). Associations between sedentary behaviour and physical activity in
children and adolescents: a meta-analysis. Obes Rev.

