# Supplementary material for: Prevalence of sedentary behavior and its correlates among primary and secondary school students
Source: Rev Paul Pediatr. 2016 Jan-Mar;34(1):56–63. doi: 10.1016/j.rppede.2015.09.002 (PMC4795722; doi:10.1016/j.rppede.2015.09.002)
Supplement: Translation

# Translation: Prevalência de comportamento sedentário de escolares e fatores associados

**Authors:** Rodrigo Wiltgen Ferreira, Airton José Rombaldi, Luiza Isnardi Cardoso Ricardo, Pedro Curi Hallal, Mario Renato Azevedo

**Affiliations:** a Programa de Pós-Graduação em Educação Física, Universidade Federal de Pelotas (Ufpel), Pelotas, RS, Brasil; b Programa de Pós-Graduação em Epidemiologia, Universidade Federal de Pelotas (Ufpel), Pelotas, RS, Brasil

**Keywords:** Estilo de vida sedentário, Comportamento do adolescente, Adolescentes, Televisão, Internet

## Abstract

**Objetivo:**

Determinar a exposição de escolares a quatro indicadores diferentes de comportamento sedentário (CS) e suas associações com gênero, série escolar, idade, condição econômica e nível de atividade física.

**Métodos:**

Um estudo transversal foi feito em 2013. Os CS foram obtidos com o instrumento Helena, composto por perguntas sobre tempo de tela (TV, videogames e internet) e atividades na posição sentada na escola em relação ao turno. O ponto de corte de ≥2 horas/dia foi usado para categorizar o desfecho. A regressão de Poisson foi usada para avaliar associações entre o desfecho e as variáveis independentes (nível de significância de 95%) e controlar as variáveis de confusão e o possível efeito do desenho.

**Resultados:**

A amostra foi composta por 8.661 alunos. A prevalência geral de CS foi de 69,2% (IC95%: 68,1-70,2) em dias de semana e 79,6% (IC95%: 78,7-80,5) nos fins de semana. O sexo feminino mostrou maior associação com o desfecho, exceto para jogos eletrônicos. Estudantes de séries mais avançadas estavam mais envolvidos em tarefas na posição sentada, quando comparados com os das séries iniciais. Os alunos mais velhos eram mais propensos a navegar na internet por mais de duas horas por dia. Estudantes com condição econômica mais elevada eram mais propensos a passar o tempo em videogames e internet. Indivíduos ativos eram menos propensos a se envolver em CS durante a semana.

**Conclusões:**

A prevalência da CS foi elevada, principalmente nos fins de semana. As associações com sexo, idade, série escolar e nível de atividade física devem ser consideradas para elaborar intervenções mais eficientes no controle dos CS.

## Introdução

Desde o fim da II Guerra Mundial, houve uma intensificação no processo de comunicação, particularmente estimulada pelo advento da televisão. A intensificação do processo de comunicação traz benefícios, mas nas últimas décadas estudos têm demonstrado que o excesso de tempo sedentário pode levar a problemas de saúde, especialmente entre as novas gerações, que crescem em uma era de uso maciço da tecnologia.^1^ O comportamento sedentário (CS) está sendo conceitualizado na literatura como qualquer atividade com um custo de energia igual ou inferior a 1,5 METs^1^ feita na posição reclinada ou sentada.^2^

A infância e a adolescência são épocas particularmente relevantes para o estudo do CS, porque o período é caracterizado por mudanças físicas e mentais acentuadas.^3^ Nesse sentido, há evidências de que o CS tem impacto direto sobre desfechos de saúde, como obesidade, síndrome metabólica e doenças cardiovasculares.^4^-^6^ Também tendo sido descrito como relacionado a reduções na expectativa de vida.^7^ Devido aos seus efeitos sobre a saúde, recomendações sobre CS foram publicadas em 2001, com uma atualização em 2011.^8^

Um recente estudo de revisão identificou 24 estudos brasileiros sobre CS, a maioria dos quais com foco em mídia digital ou tempo de tela (televisão, jogos e computador).^9^ No entanto, as diferenças entre as ferramentas de medição (estrutura do questionário), assim como as abordagens analíticas (limiares de CS, tipos de regressão e possível controle de fatores de confusão), tornam difícil a comparação de dados de diferentes estudos. Além disso, é necessário analisar as possíveis associações com variáveis sociais, demográficas e comportamentais, a fim de fazer intervenções eficazes para controlar CS.

O objetivo do presente estudo foi avaliar a exposição a quatro indicadores de CS em adolescentes da cidade de Pelotas, RS, Brasil e suas associações com sexo, série escolar, idade, nível econômico e atividade física.

## Método

Esse estudo transversal fez parte da terceira coleta de dados de acompanhamento de uma intervenção chamada Educação física +: Praticando a saúde na escola. Esse estudo foi feito em 56 escolas públicas de Pelotas em 2012 e 2013. O principal objetivo da intervenção foi disseminar informações relacionadas à atividade física e à saúde em geral por meio das aulas de educação física. Os dados apresentados neste artigo são um instantâneo da exposição ao CS.

Um processo de amostragem de múltiplos estágios foi usado e dividido em duas etapas, referentes a cada ano de intervenção. Um sorteio foi feito a cada ano entre todas as escolas elegíveis da cidade para garantir a representatividade da amostra. Mais informações sobre o processo de amostragem estão disponíveis no estudo de Spohr et al.^10^ A primeira etapa foi feita em 2012. Foi obtida uma lista das escolas públicas de ensino fundamental e médio da cidade. Então, estratificamos as escolas de acordo com o tipo (estadual *versus* municipal) e a área da cidade (urbana *versus* rural). Uma estratégia aleatória foi adotada para selecionar a escola em cada estrato, totalizando 40 escolas na amostra de 2012. No segundo ano do estudo (2013) a mesma estratégia foi adotada para a amostragem, mas outras 18 escolas foram incluídas. Duas escolas foram removidas da amostra original (sorteadas em 2012). Uma escola recusou-se a participar do estudo e outra foi excluída porque todos os alunos elegíveis pertenciam ao turno da noite. Um ponto importante é que, após a coleta de dados com início em 2013, não houve outras recusas. O número final de escolas participantes (n=56) representou 67% de todas as escolas elegíveis da cidade.

Uma versão adaptada do instrumento Helena, proposto pela primeira vez por Rey-López et al.^11^ (coeficiente Kappa >0,7), foi usado para avaliar CS. O instrumento foi traduzido para o português e, em seguida, de volta para o espanhol, a fim de garantir a clareza e o significado da informação. O CS é avaliado por meio de perguntas sobre o uso de televisão, jogos eletrônicos, internet e atividades acadêmicas no turno inverso das aulas. As perguntas são feitas primeiramente sobre os dias da semana e, em seguida, sobre os dias do fim de semana. Para quantificar a duração do CS, há uma escala de tempo na qual o entrevistado deve escolher entre sete categorias, que variam de “nenhum” até “quatro horas ou mais”’ por dia. O CS foi categorizado de acordo com as recomendações da Academia Americana de Pediatria.^8^

A coleta de dados ocorreu de março a maio de 2013. Alunos da 5° ano do Ensino Fundamental até o 3° ano do Ensino Médio foram convidados a participar do estudo. O questionário foi autoadministrado na sala de aula, sob a supervisão de um entrevistador treinado. A aplicação do questionário foi coletiva. Os estudantes responderam as perguntas após a explicação do entrevistador para cada pergunta. Se um aluno tivesse alguma dúvida, o entrevistador resolvia o caso individualmente.

As variáveis independentes usadas nesta análise foram: sexo, idade (categorizada em cinco grupos <12, 13, 14, 15, ≥16), ano escolar (5°do Ensino Fundamental ao 3° ano do Ensino Médio), nível socioeconômico e atividade física. A classificação do nível socioeconômico foi baseada em um índice de ativos mais tarde categorizados em quintis, após a análise dos componentes principais. O questionário de Farias et al.^12^ foi usado para avaliar os níveis de atividade física (validade: k=0,59 e CCI=0,88/reprodutibilidade: k=0,52 e Spearman=0,62). Esse instrumento tem uma lista de atividades físicas na qual o entrevistado deve responder sobre a frequência e duração das atividades feitas na semana anterior. Um escore total de atividade física foi calculado e, posteriormente, categorizado como cumprindo as recomendações atuais de 300min por semana ou não.^13^ Por decisão operacional, foi usada apenas a seção de atividade física no lazer. O instrumento original foi testado em escolas públicas de duas cidades próximas.

Os dados foram digitados em duplicata no programa EpiData 3.1 e as análises foram feitas com o programa Stata 12.0. A regressão de Poisson foi usada na análise ajustada para verificar a associação entre cada tipo de comportamento sedentário e as variáveis independentes, ajustada para fatores de confusão e um possível efeito do desenho. Além disso, na análise ajustada, todas as variáveis independentes, com um valor de *p*>0,20, foram excluídas do modelo e um nível de significância de 95% foi adotado para as associações entre o desfecho e as exposições.

O estudo foi aprovado pelo Comitê de Ética em Pesquisa da Escola de Educação Física da Universidade Federal de Pelotas sob o protocolo n^o^ 039/2011. O termo de consentimento por escrito foi solicitado dos pais de alunos com menos de 18 anos e diretamente dos estudantes com 18 anos ou mais.

## Resultados

A amostra foi composta por 8.661 alunos, o que representa 57,7% de todos os indivíduos elegíveis. As taxas de resposta foram de 47,6% no Ensino Médio e 59,7% no Ensino Fundamental. A maioria dos participantes era do sexo feminino (53,1%), frequentava o Ensino Fundamental (76,8%), tinham menos de 12 anos (28,6%) e era ativa no lazer (57,5%). A prevalência total de comportamento sedentário foi de 69,2% durante a semana e 79,6% nos fins de semana.

A figura 1 descreve o tempo usado para cada tipo de CS em dias de semana e fins de semana. Assistir a TV por duas ou mais horas por dia foi relatado por 40% dos adolescentes nos dias de semana e de 50% nos fins de semana. A proporção de estudantes que jogam jogos eletrônicos por duas ou mais horas por dia foi de 29% nos dias úteis e 44% nos fins de semana. Para o uso da internet, essas proporções foram de 41% e 55%, respectivamente. Passar duas ou mais horas em atividades na posição sentada no turno inverso da escola foi relatado por 18% dos entrevistados em dias de semana e por 10% nos dias de fim de semana.

**Figura 1:** Comportamento sedentário dos estudantes em dias de semana e fins de semana, Pelotas-RS Brasil, 2013, n=8.661.

A figura 2 mostra o acúmulo de CS para ≥2h/dia em diferentes tipos de comportamentos medidos. Para o período dos dias da semana, 31% da amostra acumulada ≤2h/dia, em qualquer medida de CS, 30% relataram duas horas ou mais por dia em um comportamento, 23% acumularam em dois tipos de CS, 14% em três e 2% em quatro. No fim de semana, as proporções foram de 20%, 27%, 29%, 21% e 3%, respectivamente.

**Figura 2:** Acúmulo de comportamento sedentário em duas ou mais horas por dia em diferentes indicadores de comportamento sedentário, Pelotas-RS Brasil, 2013, n=8.661.

A tabela 1 mostra a análise ajustada entre o excesso de CS (≥2h/dia) em dias de semana de acordo com as variáveis independentes. As meninas tinham mais probabilidade do que os rapazes de assistir a TV por períodos mais longos, bem como de executar tarefas na posição sentada no turno inverso da escola. Os meninos, por outro lado, eram mais propensos do que as meninas a passar duas ou mais horas por dia jogando jogos eletrônicos. Não houve diferenças entre os sexos para o uso do computador. A feitura de tarefas na posição sentada no turno inverso da escola aumentou de acordo com a série escolar. O uso de jogos eletrônicos e internet por duas ou mais horas por dia foi maior entre os adolescentes de maior nível socioeconômico em comparação com os de grupos socioeconômicos mais baixos. Adolescentes ativos eram menos propensos a ver televisão ou usar a internet por duas ou mais horas por dia, em comparação com seus pares inativos.

**Tabela 1:** Análise ajustada entre o excesso de comportamento sedentário (≥2h/dia) e variáveis sociodemográficas e comportamentais em dias de semana, Pelotas‐RS Brasil, 2013, n = 8.661

			Comportamento sedentário ≥ 2 h durante a semana														
			Televisão				Jogos eletrônicos				Internet				Atividades na posição sentada		
			%	RP (IC95%)	*p‐valor*		%	RP (IC95%)	*p‐valor*		%	RP (IC95%)	*p‐valor*		%	RP (IC95%)	*p‐valor*
**Sexo**					0,01				<0,001				0,36				0,005
	*Masculino*		38,3	1,0			37,9	1,0			39,3				16,2	1,0	
	*Feminino*		42,2	1,09 (1,01‐1,18)			21,5	0,58 (0,54‐0,63)			39,7				18,9	1,14 (1,03–1,26)	

**Série**					0,09				<0,001				0.67				<0,001
	*Ensino Fundamental*																
		5^a^	38,1	1,0			24,4	1,0			25,6				12,9	1,0	
		6^a^	39,5	0,99 (0,89‐1,12)			28,1	1,11 (0,98‐1,24)			32,2				13,3	0,98 (0.82‐1.18)	
		7^a^	44,0	1,11 (0,99‐1,24)			33,2	1,30 (1,12‐1,52)			41,9				14,9	1,03 (0.84‐1.27)	
		8^a^	41,9	0,99 (0,88‐1,11)			34,3	1,31 (1,14‐1,52)			48,4				20,6	1,38 (1,11‐1,72)	
	*Ensino Secundário*																
		1^a^	38,7	0,91 (0,80‐1,03)			31,9	1,16 (0,96‐1,40)			52,0				22,3	1,50 (1,18‐1,92)	
		2^a^	39,3	0,93 (0,81‐1,08)			26,7	1,02 (0,84‐1,23)			50,7				28,4	1,84 (1,43‐2,37)	
		3^a^	41,1	0,94 (0,77‐1,13)			22,3	0,88 (0,70‐1,11)			49,8				30,8	1,89 (1,36‐2,64)	
																	
**Idade**					0,07				0,001				<0,001				<0,001
	*≤12*		38,5	1,0			26,3	1,0			28,6	1,0			12,2	1,0	
	*13*		39,7	0,99 (0,90‐1,11)			30,3	1,02 (0,92‐1,14)			38,7	1,33 (1,19‐1,49)			16,4	1,24 (1,06‐1,44)	
	*14*		45,1	1,17 (1,07‐1,27)			31,5	1,03 (0,92‐1,16)			44,8	1,51 (1,36‐1,67)			18,4	1,25 (1,02‐1,53)	
	*15*		38,9	1,05 (0,93‐1,18)			33,5	1,11 (0,98‐1,27)			45,8	1,55 (1,39‐1,74)			17,4	1,06 (0,88‐1,29)	
	*≥16*		40,3	1,10 (0,99‐1,23)			26,9	0.97 (0,82‐1,14)			47,0	1,54 (1,40‐1,69)			26,4	1,32 (1.04‐1,68)	

**Índice de ativos (quintis)**					0,36				<0,001				<0,001				0,53
	*1 (mais baixo)*		41,6				14,9	1,0			16,1	1,0			16,5		
	*2*		41,4				26,0	1,75 (1,48‐2,08)			34,6	2,21 (1,91‐2,56)			16,1		
	*3*		38,4				30,7	2,04 (1,76‐2,38)			41,9	2,63 (2,25‐3,06)			18,7		
	*4*		41,1				34,5	2,31 (1,98‐2,70)			47,5	2,96 (2,59‐3,39)			17,8		
	*5 (mais alto)*		39,6				39,0	2,53 (2,19‐2,94)			55,9	3,47 (3,00‐4,01)			18,9		

**Atividade física**					0,02				0,35				0,003				0,39
	*<300min*		42,7	1,0			26,1				41,0	1,0			18,5		
	*≥300min*		38,6	0,93 (0,87‐0,99)			31,5				39,0	0,93 (0,89‐0,98)			17,2		

A tabela 2 mostra a análise ajustada entre o excesso de CS (≥2h/dia) nos fins de semana de acordo com as variáveis independentes. As meninas tinham mais probabilidade do que os meninos de passar duas ou mais horas em todos CS, exceto os jogos eletrônicos. Os adolescentes de séries escolares mais avançadas eram menos propensos a fazer tarefas na posição sentada nos fins de semana. Associações positivas foram confirmadas entre o nível socioeconômico e (a) jogar jogos eletrônicos e (b) o uso da internet nos fins de semana. Níveis de atividade física não foram preditores de qualquer tipo de CS nos fins de semana.

**Tabela 2:** Análise ajustada entre o excesso de comportamento sedentário (≥2h/dia) e variáveis sociodemográficas e comportamentais nos finais de semana. Pelotas‐RS Brasil, 2013, n = 8.661

			Comportamento sedentário ≥ 2 h durante a semana														
			Televisão				Jogos eletrônicos				Internet				Atividades na posição sentada		
			%	RP (IC95%)	*p‐valor*		%	RP (IC95%)	*p‐valor*		%	RP (IC95%)	*p‐valor*		%	RP (IC95%)	*p‐valor*
**Sexo**					0,007				<0,001				0,04				0,03
	*Masculino*		48,7	1,0			55,4	1,0			54,3	1,0			10,0	1,0	
	*Feminino*		51,7	1,06 (1,02‐1,11)			33,9	0,63 (0,59‐0,66)			55,2	1,04 (1,00‐1,09)			10,8	1,16 (1,02–1,32)	

**Série**					0,68				<0,001				0,83				0,04
	*Ensino Fundamental*																
		5^a^	49,2				42,4	1,0			43,4				10,9	1,0	
		6^a^	50,6				47,5	1,09 (1,02‐1,18)			51,0				9,2	0,85 (0,65–1,11)	
		7^a^	51,5				46,8	1,09 (1,00‐1,19)			55,3				9,3	0,76 (0,56–1,02)	
		8^a^	50,5				44,2	1,02 (1,93‐1,11)			61,3				9,9	0,77 (0,56–1,06)	
	*Ensino Secundário*																
		1^a^	50,1				44,4	0,97 (0,86‐1,09)			65,0				9,6	0,64 (0,45–0,90)	
		2^a^	50,1				38,6	0,85 (0,76‐0,96)			65,2				13,8	0,81 (0,56–1,16)	
		3^a^	59,4				33,3	0,74 (0,65‐0,84)			62,1				16,2	0,90 (0,63–1,29)	

**Idade**					0,30				0,94				<0.001				<0,001
	*≤12*		49,6				43,0				44,5	1,0			8,9	1,0	
	*13*		52,8				47,5				55,7	1,23 (1,15‐1,31)			8,9	1,11 (0,87–1,40)	
	*14*		51,5				44,5				61,0	1,32 (1,24‐1,40)			9,9	1,29 (1,03–1,63)	
	*15*		48,1				45,9				58,0	1,27 (1,20‐1,35)			10,3	1,40 (1,05–1,85)	
	*≥16*		48,8				39,4				60,9	1,29 (1,23‐1,37)			14,5	1,96 (1,52–2,54)	

**Índice de ativos (quintis)**					0,45				<0,001				<0,001				0,52
	*1 (Mais baixo)*		51,7				27,0	1,0			26,8	1,0			10,0		
	*2*		50,3				41,3	1,54 (1,39‐1,71)			48,9	1,84 (1,69‐2,01)			10,0		
	*3*		48,5				44,4	1,65 (1,50‐1,82)			59,1	2,20 (1,98‐2,46)			10,6		
	*4*		52,1				51,0	1,91 (1,68‐2,17)			64,9	2,40 (2,18‐2,65)			10,7		
	*5 (Mais alto)*		49,0				55,7	2,04 (1,85‐2,24)			62,1	2,70 (2,42‐3,02)			10,8		

**Atividade física**					0,21				0,43				0,44				0,14
	*<300min*		51,7				38,5				55,1				10,1	1,0	
	*≥300min*		49,3				47,7				55,0				10,3	1,07 (0,94‐1,22)	

## Discussão

Este estudo teve como objetivo avaliar a exposição à CS em uma cidade no sul do Brasil, bem como o estudo de sua associação com o sexo, série escolar, idade, nível socioeconômico e nível de atividade física. As dificuldades encontradas na literatura internacional e nacional de estabelecer a real magnitude do CS e seus fatores associados resultam de diferentes culturas/costumes empregados na pesquisa e a rotina do CS de cada população. Assim, contribuir para a compreensão do CS e seus fatores associados é importante para que as intervenções se concentrem na interação regular da população com a mídia digital, em vez de banir a tecnologia da vida das pessoas.

Houve uma clara tendência de aumento do CS em fins de semana, em comparação com os dias de semana. Dados de um estudo multicêntrico feito em sete países europeus que usaram o mesmo instrumento para avaliar o CS encontraram a mesma relação.^14^ Entretanto, um estudo de revisão sobre CS em crianças^15^ e um estudo que usou acelerometria^16^ encontraram associações no sentido inverso, o que indica a incerteza sobre a relação entre CS e fins de semana. Padrões culturais, ambientais e sociais inerentes a cada região ou país podem influenciar o CS e, talvez, explicar as diferenças entre os estudos.

Especificamente a respeito dos indicadores de CS, não há evidência sólida a respeito de tempo na tela. A prevalência de excesso de tempo assistindo a televisão no presente estudo corrobora outros estudos publicados.^17^^,^ 18 Para os comportamentos relacionados a tempo de tela restantes, a prevalência encontrada aqui também foi semelhante à dos estudos anteriores.^19^^,^ 20 Em um estudo de revisão feito por Barbosa Filho et al.^9^ foi detectada uma grande variação na prevalência de CS nos estudos. Em estudos que usaram um ponto de corte de duas horas por dia a prevalência variou de 32% em um estudo feito em Foz do Iguaçu (PR) a 88% em um estudo feito em Ouro Preto (MG).^9^

Foi possível identificar um grande acúmulo de diferentes tipos de CS para ≥2h/dia, especialmente em fins de semana. O crescente uso de mídias digitais promoveu mudanças culturais na sociedade.^1^ O fácil acesso a dispositivos eletrônicos, as modificações nos ambientes familiares e a falta de segurança da vizinhança fazem com que as crianças permaneçam por mais tempo reclusas em suas casas, um fato que pode promover uma maior exposição ao CS.^21^ Além disso, o uso de mais de um dispositivo eletrônico simultaneamente é cada vez mais comum entre os jovens, o que aumenta o CS nessa população.^22^^,^ 23

Meninas foram mais propensas a exceder a quantidade diária recomendada de CS nos dias de semana e fins de semana em quase todos os tipos de CS, exceto “jogar jogos eletrônicos”. A associação entre o CS e o sexo ainda não é um consenso na literatura.^6^^,^ 24 O estudo de Atkin et al.^25^ que usou acelerometria para medir CS demonstrou que a frequente restrição às atividades ao ar livre imposta pelos pais estava associada com um aumento no tempo de sedentarismo de suas filhas após um ano de seguimento.

Idade foi associada de forma positiva com o uso da internet em nossa amostra. Um estudo que avaliou o uso compulsivo de internet pelos jovens também demonstrou uma relação direta com a idade.^26^ As diferentes maneiras de usar a internet e sua interatividade com a vida diária a tornam uma prática versátil e culturalmente aceita, com uma forte tendência para intensificar-se ainda mais no futuro próximo.^27^

Especificamente em relação a associações entre série escolar e tipos de CS, houve uma relação inversa entre o tempo gasto em jogos eletrônicos e estar envolvido em atividades na posição sentada no turno inverso da escola em dias de semana. O aumento das responsabilidades ao longo dos anos pode ser um fator importante nessa associação. A busca por bons desempenhos em processos de seleção e uma busca maior por melhoria para conquistar um lugar no mercado de trabalho são comuns entre os adolescentes,^28^ o que pode explicar a redução do tempo disponível para CS quando há atividades no contraturno.

Quanto à relação entre o nível econômico e CS, houve uma relação direta entre eles nos dias de semana e fins de semana para todos os comportamentos relacionados ao uso de tela. Nossas associações entre nível socioeconômico e CS foram consistentes com estudos anteriores. O aumento do poder aquisitivo familiar é um fator importante de CS em crianças e adolescentes.^5^^,^ 19 ^,^ 29

Uma recente metanálise feita por Pearson et al.^30^ resumiu os resultados de 163 estudos publicados desde agosto de 2013 sobre a associação entre CS e atividade física. A revisão mostrou uma associação inversa, mas de magnitude fraca. Aqui encontramos associações inversas para o ato de assistir a TV e uso de internet, de cerca de 7%.

O presente estudo tem algumas limitações. Em primeiro lugar, as exposições e os resultados foram baseadas em autorrelato. A natureza transversal dos dados torna impossível estudar a temporalidade das associações entre CS e atividade física. A taxa de resposta relativamente baixa, especialmente para o ensino secundário, também pode afetar nossos resultados.

Em conclusão, a prevalência da CS na população estudada foi alta, especialmente nos fins de semana. CS parece mais evidente nos indivíduos de maior nível socioeconômico e em meninas, com exceção dos jogos eletrônicos. Além disso, indivíduos pertencentes às séries escolares mais avançadas e os mais velhos parecem ser mais propensos a se envolver em tarefas sedentárias no turno inverso da escola e a usar a Internet. Futuros estudos sobre CS devem levar em consideração esses resultados, devido à influência de fatores associados, como idade, sexo, série escolar e atividade física, sobre as estratégias das intervenções. Além disso, é necessário que os profissionais das áreas de saúde e educação entendam a importância de alertar os jovens e seus cuidadores sobre os riscos de uma rotina excessivamente sedentária.

## Footnotes

1 Unidade de intensidade de atividade física referente ao metabolismo basal. Um MET é equivalente a 3,5mL/kg/min. ou 1kcal/kg/h. Por exemplo, caminhadas vigorosas requerem quatro vezes mais energia do que o metabolismo basal, portanto, 4 METs. O fato de ficar na posição em pé requer duas vezes mais energia do que o metabolismo basal (2 METs), então, o indivíduo não é considerado em CS.
